# BioContainers Registry: searching bioinformatics and proteomics tools, packages, and containers

**DOI:** 10.1021/acs.jproteome.0c00904

**Published:** 2021-02-24

**Authors:** Jingwen Bai, Chakradhar Bandla, Jiaxin Guo, Roberto Vera Alvarez, Mingze Bai, Juan Antonio Vizcaíno, Pablo Moreno, Björn Grüning, Olivier Sallou, Yasset Perez-Riverol

**Affiliations:** 1 European Molecular Biology Laboratory, European Bioinformatics Institute (EMBL-EBI), Wellcome Trust Genome Campus, Hinxton, Cambridge, CB10 1SD, UK; 2 College of Bioinformation, Chongqing University of Posts and Telecommunications, Chongqing, 400065, China; 3 Computational Biology Branch, National Center for Biotechnology Information, National Library of Medicine, National Institutes of Health, Bethesda, MD, USA; 4 Chongqing Key Laboratory of Big Data for Bio Intelligence, Chongqing, 400065, China; 5 Bioinformatics Group, Department of Computer Science, University of Freiburg, Freiburg,79110, Germany; 6 Institut de Recherche en Informatique et Systèmes Aléatoires (IRISA/INRIA) -GenOuest Platform, Université de Rennes, Rennes, France

**Keywords:** BioContainers, Cloud, High-performance computing, Large-scale data analysis, Computational proteomics

## Abstract

BioContainers is an open-source project that aims to create, store, and distribute bioinformatics software containers and packages. The BioContainers community has developed a set of guidelines to standardize the software containers including the metadata, versions, licenses, and/or software dependencies. BioContainers supports multiple packaging and container technologies such as Conda, Docker, and Singularity. The BioContainers provide over 9000 bioinformatics tools including more than 200 proteomics and mass spectrometry tools. Here, we introduce the BioContainers Registry and Restful API to make containerized bioinformatics tools more findable, accessible, interoperable, and reusable (FAIR). The BioContainers Registry provides a fast and convenient way to find and retrieve bioinformatics tools packages and containers. By doing so, it will increase the use of bioinformatics packages and containers while promoting replicability and reproducibility in research.

## Introduction

The BioContainers community (https://biocontainers.pro) has created a complete ecosystem that enables bioinformatics software to be installed and executed in an isolated and controlled environment ^1,2^. Also, it provides infrastructure and basic guidelines to create and distribute bioinformatics containers focusing especially on omics technologies. By January 2021, BioContainers provides over 9,000 tools, 29,000 software versions, and 130,000 packages and containers. It gives access to containers for multiple technologies including Conda ^3^, Docker, and Singularity ^4^. By supporting multiple packaging and container technologies, BioContainers allows running bioinformatics, and proteomics tools in proteomics tools in particular, in different architectures such as Cloud, High-performance computing clusters (HPC), or users computers.

The FAIR Guiding Principles for scientific data management provide recommendations on how to make research data findable, accessible, interoperable, and reusable (FAIR) ^5^. In 2017, Jimenez et al. ^6^ proposed that the FAIR principles for the software involved: i) Findable: the software should be easy to discover by providing software metadata such as title, general description, publication, license, and versions; ii) Accessible: the link to the code or binaries should be available; iii) Interoperable: the metadata should be exchangeable between major software registries; and iv) Reusable: involving the adoption of a license, helping to define to which extent the community can reuse the tool. By 2020, more than 200 proteomics tools were included in BioContainers, making it difficult to search, find, and in some cases know which tool is supported for your infrastructure (e.g., Cloud, HPC, or local computer).

Here, we introduce the BioContainers Registry (https://biocontainers.pro/registry) and Restful API - https://api.biocontainers.pro/ga4gh/trs/v2/ui/) to make bioinformatics, and proteomics tools in particular, more findable, accessible, interoperable, and reusable (FAIR). The Registry web interface allows bioinformaticians researchers to search for bioinformatics/proteomics tools and their corresponding packages and containers. The Restful API allows developers to programmatically interact with the BioContainers Registry.

## Materials

### From tools to packages and containers

One of the challenges of bioinformatics is to be able to install, configure, and deploy exiting tools and workflows associated with publications. Even if source code and data (of the research) are published in a public repository, the tool may have non-obvious dependencies on other software, configuration options, operating systems, and other subtleties that hamper re-usability ^3,7^. Additionally, workflows and pipelines commonly combine software developed by different groups, adding another layer of complexity and introducing challenges such as compatibility and management of dependencies ^8^.

Software package and containers have emerged as powerful technology to address primary dependency issues and enable distributing and deploying scientific software in a runnable state. Conda manages and distributes software packages in an isolated Python environment, transforming them into relocatable binaries ^3^. Bioconda packages are Conda packages provided and maintained by the Bioconda community ^5^. Conda keeps track of the dependencies between packages and platforms. The Conda package format is identical across platforms and operating systems ^3^. A higher level of virtualization is achieved with Docker, where tools containers are distributed with dependencies in a self-contained environment ^6,7,9^. Both technologies (Conda and Docker) are suitable for different architectures. Conda is well-established in HPC architectures where virtualization is not highly demanded, and the clusters can be set up with a Conda environment. In contrast, Docker is used in cloud infrastructures where virtualization is extensively used, and resources are allocated/released dynamically. However, Docker is not suitable for HPC clusters because users can root inside the container and for example, mount paths from the host that they normally don’t have access. Singularity ^4^ is to support existing and traditional HPC resources as easily as installing a single package onto the host operating system.

BioContainers infrastructure builds, stores, and releases packages and containers from three different technologies: Conda, Docker, and Singularity. The same tool is encapsulated and provided for the tree technologies enabling users to use the tool in personal computers, HPC, or cloud infrastructures.

### BioContainers infrastructure

**Figure 1:**
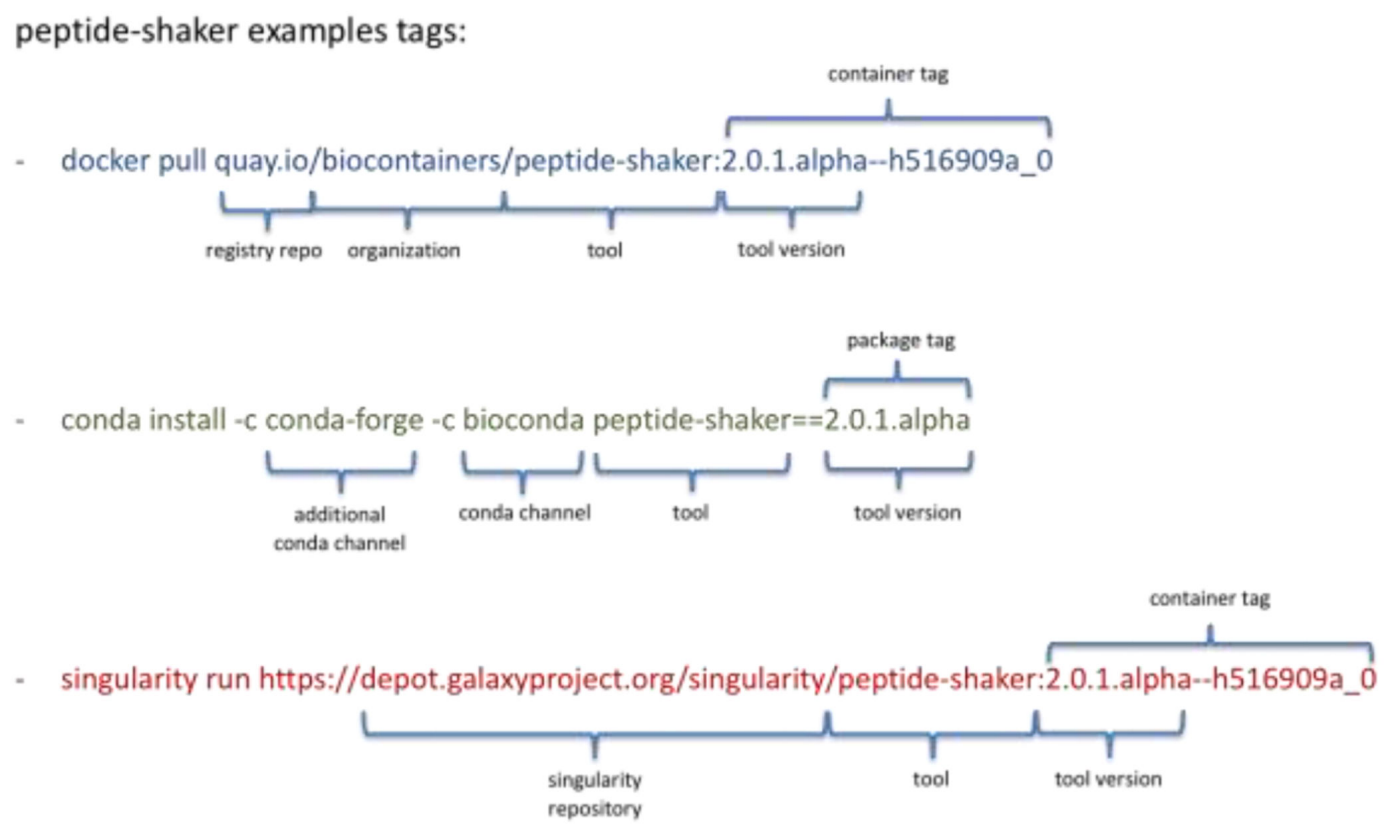
Docker, Conda, and Singularity tags available for the peptide-shaker tool. The tag is a unique identifier for each registry to find and retrieve a package or container. The full tag is the combination of the repository or registry, the tool name, and the container/package tag.

For every bioinformatics and proteomics software, a Bioconda recipe can be created and the corresponding Conda package built ^3^. The new package will be added to the Bioconda channel and the user will be ready to use it in a Conda environment. For example, the Conda package for peptide-shaker (https://biocontainers.pro/tools/peptide-shaker) can be installed using the following command*: conda install -c conda-forge -c bioconda peptide-shaker==2.0.1.alpha* (Figure 1). Then, the BioContainers infrastructure automatically built a Docker container based on the Conda package and make it available through multiple Docker registries (e.g., quay.io). For example, the Docker container for peptide-shaker can be retrieved using the following command: *docker pull quay.io/biocontainers/peptide-shaker:2.0.1.alpha--h516909a_0* (Figure 1). In addition to the Docker container, a Singularity container is automatically released and deposited in multiple Singularity registries (e.g., https://depot.galaxyproject.org/singularity/). Singularity ^4^ has recently emerged to provide ease non-root access containers in HPC solutions. Singularity images can be run using the following command: *singularity run* https://depot.galaxyproject.org/singularity/peptide-shaker:2.0.1.alpha--h516909a_0 (Figure 1). While the BioContainers community recommends, as a rule, one tool, one container ^1^, the BioContainers infrastructure allows the creation of multitools containers from multiple Conda packages. The multitools containers are useful when a workflow used multiple tools and the users want to distribute all of them merge into a single container ^7^. Multiple manuscripts from both communities (BioContainers and Conda) previously explained how containers are created and how they can be used in combination with bioinformatics workflows ^1–3,7,8,10^.

Packages and containers are stored and can be retrieved from multiple registries depending on the particular technology (e.g., Conda, Docker, and Singularity). The BioContainers community use multiple registries (Supplementary Figure 1) for docker (DockerHub, quay.io, Elixir registry), one registry for Conda packages (Anaconda), and two repositories for Singularity images (Galaxy depo, Elixir singularity repo). The use of multiple endpoints for the same technology (e.g., Docker) acts as a failover system; when one registry is down, the other registry can serve the containers. To centralize the search across all these registry endpoints; the BioContainers infrastructure stores all the metadata from all the tools and containers in a MongoDB and provides a Registry and Restful API to facilitate search and retrieval of the bioinformatics and proteomics tools (Supplementary Figure 1).

### BioContainers registry

**Figure 2:**
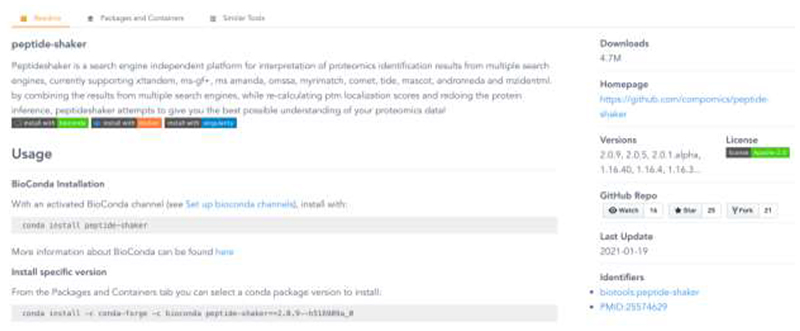
The BioContainers Registry page for the peptide-shaker tool. The readme panel contains information about the tool including a general description, license, tool home page, versions, and external identifiers (e.g., PubMed). The full-page is a general description of how to install and update the specific tool for each of the provided technologies: Conda, Docker, or Singularity.

The BioContainers Registry (https://biocontainers.pro/registry) provides an easy-to-use interface for users to search and retrieve the bioinformatics tools and the corresponding containers. The search page contains a *search box* where users can search for the required tool using keywords. The search results are then displayed as small boxes, where the name, description, license, and number of downloads are shown. Users can sort using the number of downloads/pulls and filter the results using tool tags and licenses. Figure 2 shows a tool page that describes a selected bioinformatics tool (e.g., peptide-shaker). The readme tab (Figure 2) shows the general information about the tool including steps for installation, update, and how to run. In addition, the list of versions, the last update date of the tool, the number of downloads, and all the additional identifiers (e.g. PubMed, and bio.tools ^11^ identifiers) are shown. In the example (Figure 2), peptide-shaker has been pulled from BioContainers repositories more than 3.6 million times, which can be used as an additional metric ^6,11,12^ of the usability of particular bioinformatics or proteomics tool in Cloud and HPC environments.

**Figure 3:**
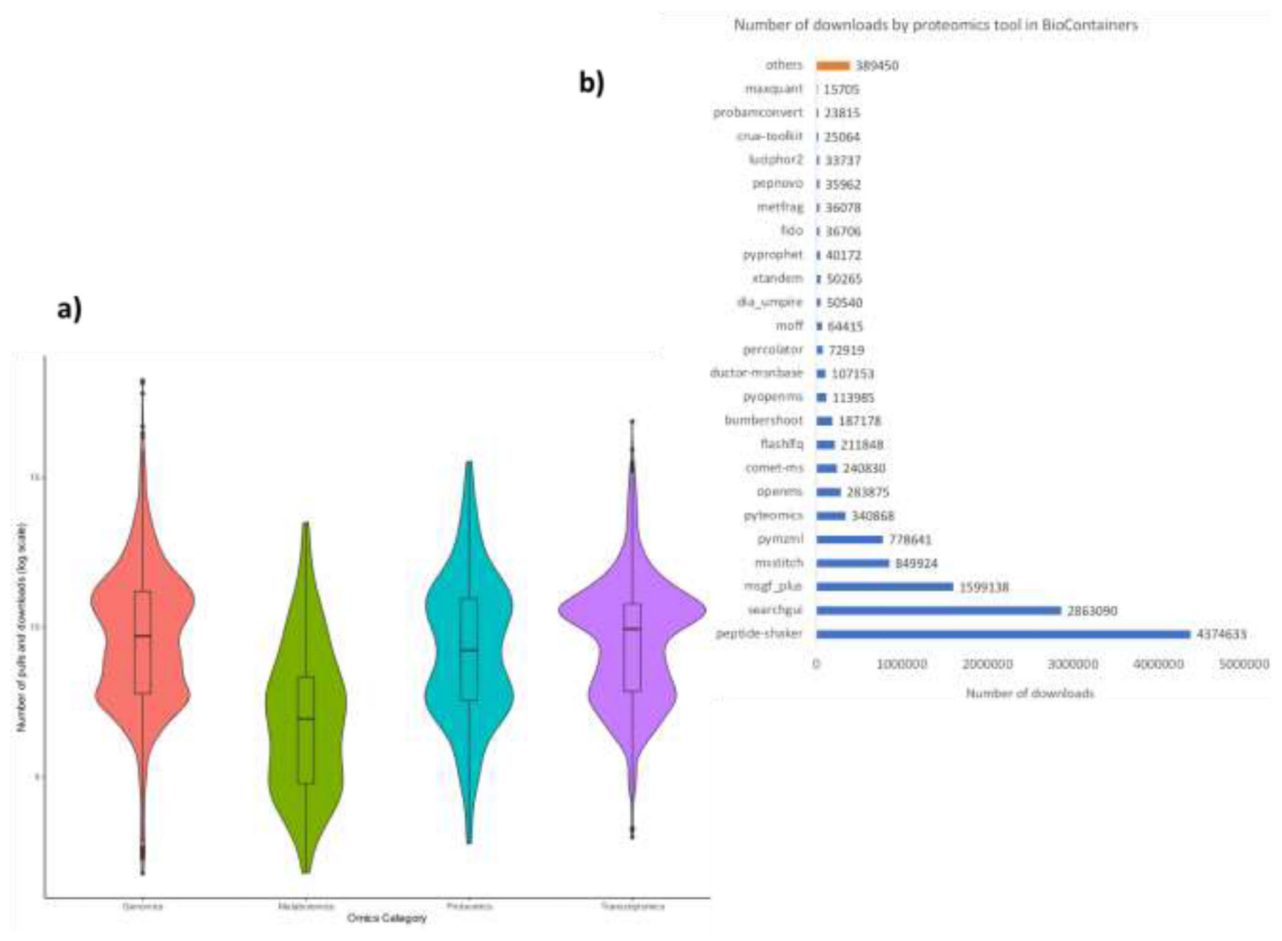
**a)** Distribution of per-container downloads (log scale), separated by specific omics category: Transcriptomics, Proteomics, Metabolomics and Genomics. Black dots in the extreme of every plot represent outliers, dark bars represent the interval between upper and lower quartiles. **b)** List of most downloaded proteomics tools from BioContainers, the term others group more than 200 proteomics tools in the registry not included in the plot.

In general, containers and packages in genomics and transcriptomics are used more frequently than proteomics and metabolomics (Figure 3a). Different from transcriptomics and genomics where some tools are extensively used compare with the rest of the bioinformatics tools (e.g., pysam or Picard), in proteomics tools usage is more uniform (Figure 3a). Figure 3b shows the most pulled and downloaded proteomics tools in BioContainers. These tools are used in combinations with workflows such as Galaxy ^13^, Nextflow ^14^, or Snakemake ^15^ which probably are the reason why they are more used than other more windows-based tools such as MaxQuant or Skyline. By adding a proteomics tool into BioContainers, developers boost the usage of their tool and the integration with existing workflow systems. A good example is MaxQuant, where the recent addition of the Linux version of the tool to BioContainers (https://biocontainers.pro/tools/maxquant) increased the usability and downloads to the tool to 11 thousand times since October 2018.

In addition to the tool description and the corresponding containers, for every tool, the registry interface provides a list of similar tools that can be used to perform the analysis (Supplementary Figure 2). To enable this, a cosine-similarity algorithm has been implemented using all the metadata available from each tool and container. In the example (Supplementary Figure 2), a list of tools including searchgui, comet, proteinphrophet is suggested to the user.

### Restful API

The BioContainers Restful API (https://api.biocontainers.pro/ga4gh/trs/v2/ui/) enables two main functionalities: (i) Search for bioinformatics tools and the corresponding containers; (ii) and retrieve the specific tool information and the corresponding containers. The search results can be sorted by the id, name, organization, description of the tool, the number of downloads, and/or the usage of each tool (Supplementary Figure 3). For example, the user can retrieve all the proteomics tools sorted by the number of downloads and pulls using the following query: https://api.biocontainers.pro//ga4gh/trs/v2/tools?all_fields_search=proteomics&sort_order=desc&sort_field=pulls.

Importantly, the Restful API is implemented following the Tool Registry Service (TRS) standard for sharing and distribute bioinformatics tools developed by The Global Alliance for Genomics and Health (GA4GH) ^16^. The TRS specification defines a standard API to exchange bioinformatics tools, workflows, and containers enabling distribution data processing of omics datasets. Multiple services in Genomics such as Dockstore (https://dockstore.org/) or Agora (https://github.com/broadinstitute/agora) distributed their tools and workflows using the specification ^16^.

### Restful API command-line interface

A python package named *bioconda2biocontainer* (https://github.com/BioContainers/bioconda2biocontainer) was developed to allow users to search from the command-line for BioContainers (*biocontainers-search*) and retrieve for a particular Conda package the particular Docker containers (*bioconda2biocontainer*). The *biocontainers-search* inquire the BioContainers Registry and return a TAB separated table with Name, Versions (comma-separated), description, license, and the number of pulls: *biocontainers-search --search_term proteomics.* The json parameter can be used to output the results in json format instead of a tab-delimited output. The name of the tool can be then provided to the *bioconda2biocontainers* tool to find the Bioconda, Docker, or Singularity containers available for the tool using the following command: *bioconda2biocontainer --package_name peptide-shaker --package_version 2.27.0 --all* (output can be seen in Supplementary Figure 4).

## Conclusion

Computational proteomics and mass spectrometry are increasingly moving from desktop applications to distributed architectures like HPC or Cloud, due to the scale of data and the complexity of the analyses. While most of the commercial tools such as ProteomeDiscover and Spectronaut remains in Windows, other popular open-source and non-commercial like MaxQuant, OpenMS or Skyline are now cross-platforms. However, to reduce the complexity of installation, deployment, and integration in complex workflows, a Bioconda package, and Docker container should be provided for every tool. BioContainers is a growing community of bioinformaticians and software developers with multiple ongoing projects, including the maintenance and creation of new software containers for bioinformatics tools, the implementation of improvements in the findability of the tools, and facilitating their re-usability. Here, we have introduced a new Restful API and web application that can be used to search for bioinformatics, and more specifically, proteomics tools, and their corresponding packages and containers. Researchers can find which are the most downloaded and used tools for a particular task, in addition to having access to all versions of a specific tool. The Restful API is an implementation of the GA4HG standard which enables compatibility with other resources and initiatives like Dockstore. The creation and reuse of software packages and containers in computational proteomics can a challenge for researchers with no bioinformatics training. The BioContainers community will help to cross this learning curve using different materials available in the help pages (http://biocontainers-edu.biocontainers.pro/en/latest/).

## Supplementary Material

Supplementary Notes

## Abbreviations

API: Application Programming Interface
FAIR: Findable, Accessible, Interoperable and Reusable
GA4GH: Global Alliance for Genomics and Health
HPC: High-Performance Computing
TRS: Tool Registry Service
