# Supplementary material for: BioContainers Registry: searching bioinformatics and proteomics tools, packages, and containers
Source: J Proteome Res. Author manuscript; Available in PMC 2021 Aug 24. (PMC7611561; doi:10.1021/acs.jproteome.0c00904)
Supplement: Supplementary Notes [file EMS133030-supplement-Supplementary_Notes.docx]

**Supplementary Information**

BioContainers Registry: searching bioinformatics and proteomics tools, packages, and containers

Jingwen Bai ^1^, Chakradhar Bandla ^1^, Jiaxin Guo ^2^, Roberto Vera Alvarez ^3^, Mingze Bai ^4^, Juan Antonio Vizcaíno ^1^, Pablo Moreno ^1^, Björn Grüning ^5, *^, Olivier Sallou ^6, *^ and Yasset Perez-Riverol ^1, *^

^1^ European Molecular Biology Laboratory, European Bioinformatics Institute (EMBL-EBI), Wellcome Trust Genome Campus, Hinxton, Cambridge, CB10 1SD, UK.

^2^ College of Bioinformation, Chongqing University of Posts and Telecommunications, Chongqing, 400065, China.

^3^ Computational Biology Branch, National Center for Biotechnology Information, National Library of Medicine, National Institutes of Health, Bethesda, MD, USA.

^4^ Chongqing Key Laboratory of Big Data for Bio Intelligence, Chongqing, 400065, China.

^5^ Bioinformatics Group, Department of Computer Science, University of Freiburg, Freiburg,79110, Germany.

^6^ Institut de Recherche en Informatique et Systèmes Aléatoires (IRISA/INRIA) -GenOuest Platform, Université de Rennes, Rennes, France.

Corresponding authors: Yasset Perez-Riverol ([yperez@ebi.ac.uk](mailto:yperez@ebi.ac.uk)), Oliver Sallou ([olivier.sallou@irisa.fr](mailto:olivier.sallou@irisa.fr)), Björn A. Grüning ([bjoern.gruening@gmail.com](mailto:bjoern.gruening@gmail.com))

Table of Contents

[Supplementary Figure 1: Full URI available for each package technology: Docker, Conda, and Singularity. The full tag is the combination of the repository or registry, the tool name, and the container/package tag. S-3](#_Toc62132180)

[Supplementary Figure 2: Similar tools and containers to peptide-shaker (https://biocontainers.pro/tools/peptide-shaker) S-4](#_Toc62132181)

[Supplementary Figure 3: Rest API call for proteomics (https://api.biocontainers.pro//ga4gh/trs/v2/tools?offset=0&limit=100&all_fields_search=proteomics&sort_field=pulls&sort_order=desc) S-5](#_Toc62132182)

[Supplementary Figure 4: Output of all packages and versions for peptide-shaker in BioContainers. S-6](#_Toc62132183)

# **Supplementary Figure 1**: Full URI available for each package technology: Docker, Conda, and Singularity. The full tag is the combination of the repository or registry, the tool name, and the container/package tag.


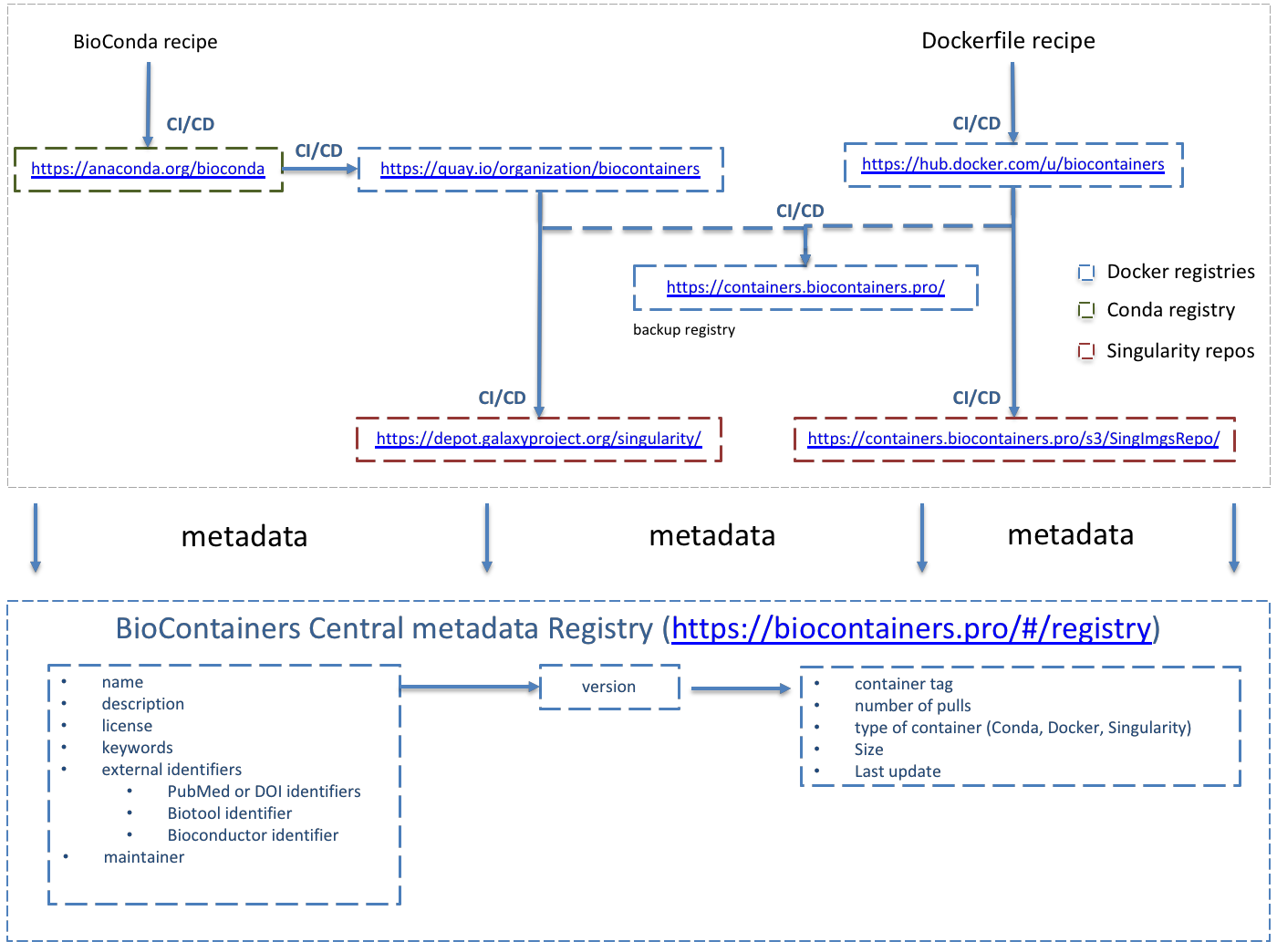


# **Supplementary Figure 2:** Similar tools and containers to peptide-shaker (<https://biocontainers.pro/tools/peptide-shaker>)

**
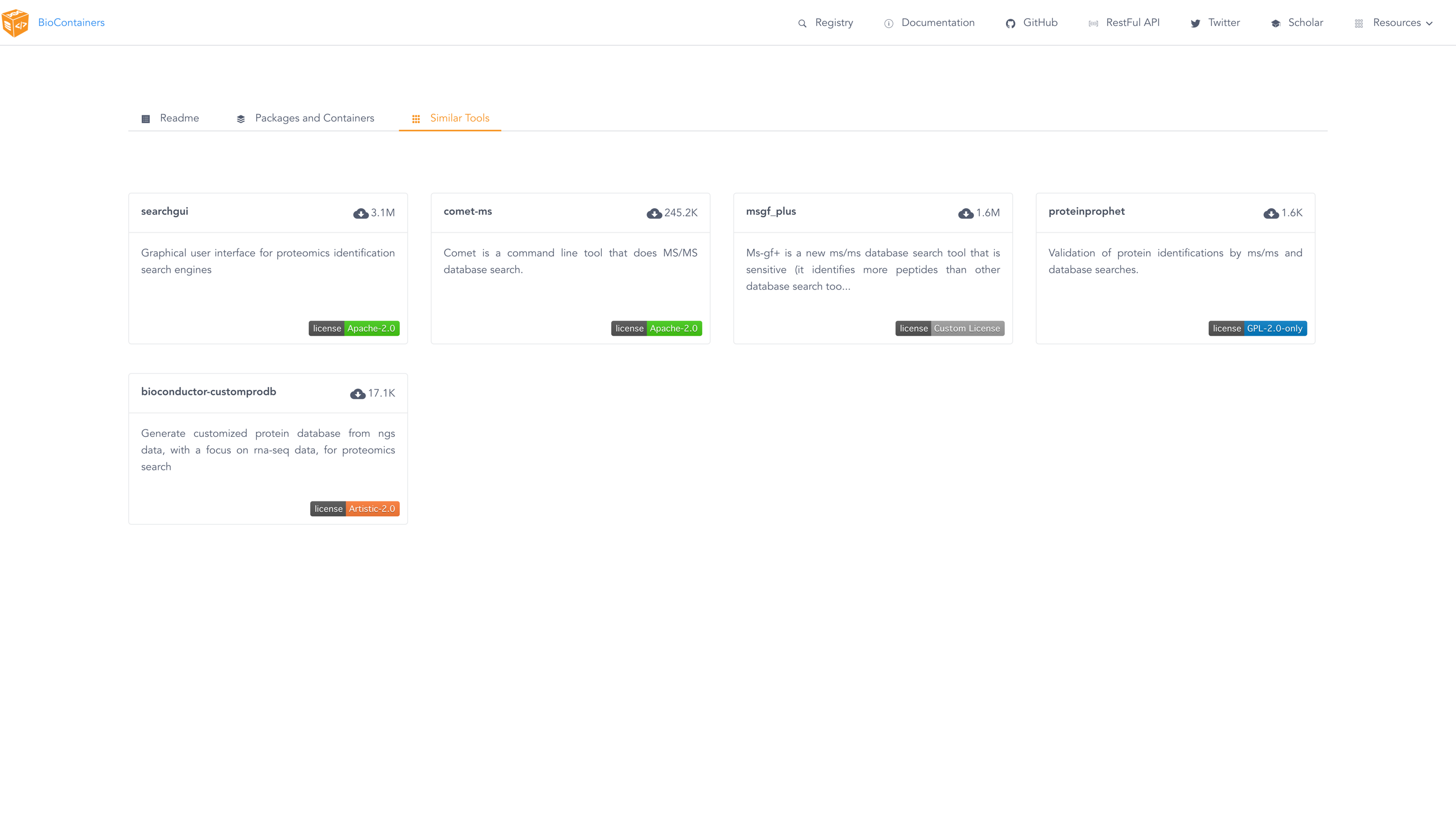
**

# **Supplementary Figure 3**: Rest API call for proteomics (<https://api.biocontainers.pro//ga4gh/trs/v2/tools?offset=0&limit=100&all_fields_search=proteomics&sort_field=pulls&sort_order=desc>)


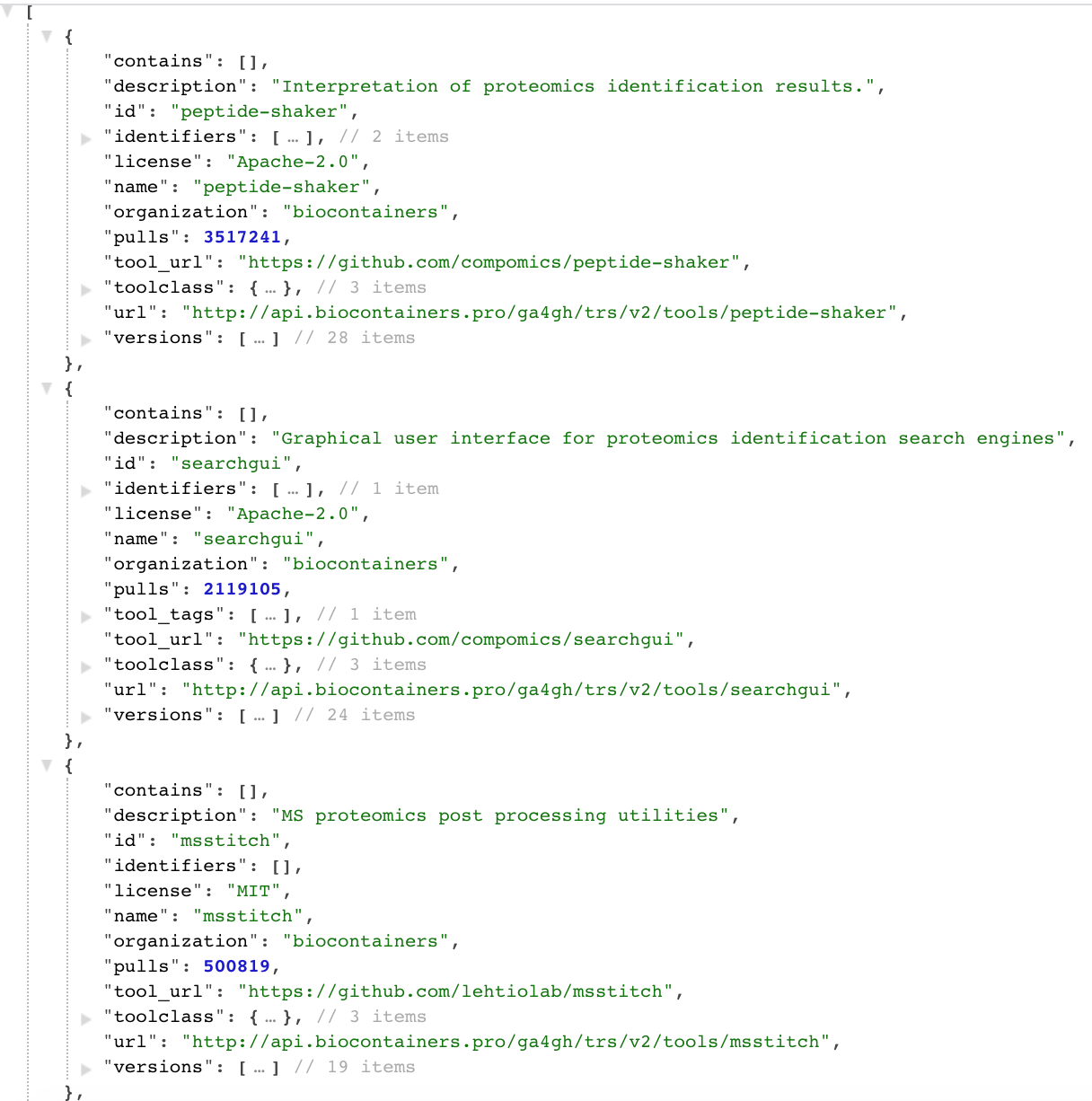


# **Supplementary Figure 4**: Output of all packages and versions for peptide-shaker in BioContainers.

**id version url**

peptide-shaker-2.0.1.alpha 2.0.1.alpha http://api.biocontainers.pro/ga4gh/trs/v2/tools/peptide-shaker/versions/peptide-shaker-2.0.1.alpha

peptide-shaker-1.16.40 1.16.40 http://api.biocontainers.pro/ga4gh/trs/v2/tools/peptide-shaker/versions/peptide-shaker-1.16.40

peptide-shaker-1.16.4 1.16.4 http://api.biocontainers.pro/ga4gh/trs/v2/tools/peptide-shaker/versions/peptide-shaker-1.16.4

peptide-shaker-1.16.36 1.16.36 http://api.biocontainers.pro/ga4gh/trs/v2/tools/peptide-shaker/versions/peptide-shaker-1.16.36

peptide-shaker-1.16.35 1.16.35 http://api.biocontainers.pro/ga4gh/trs/v2/tools/peptide-shaker/versions/peptide-shaker-1.16.35

peptide-shaker-1.16.32 1.16.32 http://api.biocontainers.pro/ga4gh/trs/v2/tools/peptide-shaker/versions/peptide-shaker-1.16.32

peptide-shaker-1.16.31 1.16.31 http://api.biocontainers.pro/ga4gh/trs/v2/tools/peptide-shaker/versions/peptide-shaker-1.16.31

peptide-shaker-1.16.30 1.16.30 http://api.biocontainers.pro/ga4gh/trs/v2/tools/peptide-shaker/versions/peptide-shaker-1.16.30

peptide-shaker-1.16.3 1.16.3 http://api.biocontainers.pro/ga4gh/trs/v2/tools/peptide-shaker/versions/peptide-shaker-1.16.3

peptide-shaker-1.16.29 1.16.29 http://api.biocontainers.pro/ga4gh/trs/v2/tools/peptide-shaker/versions/peptide-shaker-1.16.29

peptide-shaker-1.16.26 1.16.26 http://api.biocontainers.pro/ga4gh/trs/v2/tools/peptide-shaker/versions/peptide-shaker-1.16.26

peptide-shaker-1.16.23 1.16.23 http://api.biocontainers.pro/ga4gh/trs/v2/tools/peptide-shaker/versions/peptide-shaker-1.16.23

peptide-shaker-1.16.22 1.16.22 http://api.biocontainers.pro/ga4gh/trs/v2/tools/peptide-shaker/versions/peptide-shaker-1.16.22

peptide-shaker-1.16.20 1.16.20 http://api.biocontainers.pro/ga4gh/trs/v2/tools/peptide-shaker/versions/peptide-shaker-1.16.20

peptide-shaker-1.16.17 1.16.17 http://api.biocontainers.pro/ga4gh/trs/v2/tools/peptide-shaker/versions/peptide-shaker-1.16.17

peptide-shaker-1.16.16 1.16.16 http://api.biocontainers.pro/ga4gh/trs/v2/tools/peptide-shaker/versions/peptide-shaker-1.16.16

peptide-shaker-1.16.15 1.16.15 http://api.biocontainers.pro/ga4gh/trs/v2/tools/peptide-shaker/versions/peptide-shaker-1.16.15

peptide-shaker-1.16.14 1.16.14 http://api.biocontainers.pro/ga4gh/trs/v2/tools/peptide-shaker/versions/peptide-shaker-1.16.14

peptide-shaker-1.16.13 1.16.13 http://api.biocontainers.pro/ga4gh/trs/v2/tools/peptide-shaker/versions/peptide-shaker-1.16.13

peptide-shaker-1.16.0 1.16.0 http://api.biocontainers.pro/ga4gh/trs/v2/tools/peptide-shaker/versions/peptide-shaker-1.16.0

peptide-shaker-1.15.1 1.15.1 http://api.biocontainers.pro/ga4gh/trs/v2/tools/peptide-shaker/versions/peptide-shaker-1.15.1

peptide-shaker-1.15.0 1.15.0 http://api.biocontainers.pro/ga4gh/trs/v2/tools/peptide-shaker/versions/peptide-shaker-1.15.0

peptide-shaker-1.14.6 1.14.6 http://api.biocontainers.pro/ga4gh/trs/v2/tools/peptide-shaker/versions/peptide-shaker-1.14.6

peptide-shaker-1.14.4 1.14.4 http://api.biocontainers.pro/ga4gh/trs/v2/tools/peptide-shaker/versions/peptide-shaker-1.14.4

peptide-shaker-1.13.6 1.13.6 http://api.biocontainers.pro/ga4gh/trs/v2/tools/peptide-shaker/versions/peptide-shaker-1.13.6

peptide-shaker-1.13.3 1.13.3 http://api.biocontainers.pro/ga4gh/trs/v2/tools/peptide-shaker/versions/peptide-shaker-1.13.3

peptide-shaker-1.11.0 1.11.0 http://api.biocontainers.pro/ga4gh/trs/v2/tools/peptide-shaker/versions/peptide-shaker-1.11.0

peptide-shaker-1.1.3 1.1.3 http://api.biocontainers.pro/ga4gh/trs/v2/tools/peptide-shaker/versions/peptide-shaker-1.1.3
